# Supplementary material for: Tackling Prostate Cancer with Theranostic E5B9-Bombesin Target Modules (TMs): From Imaging to Treatment with UniCAR T-Cells
Source: Int J Mol Sci. 2025 Mar 17;26(6):2686. doi: 10.3390/ijms26062686 (PMC11941939; doi:10.3390/ijms26062686)
Supplement: Supplementary file 1 [file ijms-26-02686-s001.zip › ijms-3490646-supplementary.pdf]

Tackling prostate cancer with theranostic E5B9-bombesin target modules (TMs): from imaging to treatment with UniCAR T-cells

Liliana R. Loureiro, Susan Pike, Melinda Wuest, Cody N. Bergman, Kira R. JØrgensen, Ralf Bergmann, Anja Feldmann, Frank Wuest, Michael Bachmann

SUPPLEMENTARY MATERIALS

Supplementary Materials and Methods

HPLC system and methods

Semipreparative HPLC was performed on a Gilson system (Mandel Scientific, Guelph, ON, Canada) with a 321 pump and a 155 dual wavelength detector installed with a Phenomenex Jupiter 10u Proteo 90A, 250 × 10 mm, 4.5 μm C12 column. UV absorbance was monitored at wavelengths of 210 and 254 nm. The mobile phase consisted of water/0.2%TFA as solvent A and acetonitrile as solvent B.

HPLC Method 1: 2 mL/min, 0-5 min 20% B, 10 min 35% B, 30-40 min 70% B.

HPLC Method 2: 2 mL/min, 0−10 min 10% B, 25 min 50% B, 30-40 min 80% B, 40-45 min 90% B.

HPLC Method 3: 2 mL/min, 0−5 min 10% B, 10 min 30% B, 25 min 50% B, 30-40 min 80% B.

Analytical HPLC was performed on a Shimadzu system (Mandel Scientific, Guelph, ON, Canada) equipped with a DGU-20A5 degasser, an SIL-20A HT autosampler, an LC-20AT pump, an SPD-M20A photodiode array detector, and a Ramona Raytest radiodetector using a Phenomenex Luna 10u C18(2) 100A, 250 × 4.6 mm column with water/0.2% TFA as solvent A and acetonitrile as solvent B.

HPLC Method A-1: 1mL/min, 0−3 min 10% B, 10 min 30% B, 17 min 50% B, 23 min 70% B, and 27−30 min 90% B.

Synthesis of E5B9-BBN2 monomeric and dimeric TMs

Ava-Lys(N_3)-BBN2 (H_2N-Ava-Lys(N_3)-Gln-Trp-Ala-Val-Sar-His-FA01010-Tle-NH_2). Ava-Lys(N_3)-BBN2 was synthesized according to the general peptide synthesis procedure starting from 50 mg Rink Amide MBHA resin with Ava being 5-aminovaleric acid. HPLC purification (Method 1: t_R = 21.7 min) and subsequent lyophilization gave the peptide as a white solid (21.5 mg, 17.7 µmol, isolated yield: 45.3%). HPLC (Method A-1): t_R = 18.0 min, purity: 90%. MW $C_{58}H_{92}N_{18}O_{11}$ 1216.72, measured ESI-MS (positive) m/z = 1217.5 $[M+H]^+$, 609.4 $[M+2H]^{2+}$.

NOTA-Ava-Lys(N_3)-BBN2. An amount of 10 mg (1 eq., 8.2 µmol) Ava-Lys(N_3)-BBN2 and 5.5 mg (1.2 eq., 9.8 µmol) *p*-SCN-Bn-NOTA was dissolved in 300 µL of DMF in a LoBind Eppendorf tube. The pH was adjusted to 9 by the addition of 17.2 µL (123 µmol) of 15 eq. triethylamine (TEA). The reaction mixture was incubated at 25°C overnight before it was subjected to semipreparative HPLC purification (Method 2: t_R (NOTA-Ava-Lys(N_3)-BBN2) = 32.1 min). HPLC solvent was reduced under vacuum using a rotary evaporator. Lyophilization gave 9.3 mg (55.8 µmol, 68% isolated yield) of the chelator-modified peptide as a TFA salt (white powder). HPLC-QC (Method A-1): t_R (NOTA-Ava-Lys(N_3)-BBN2) = 19.2 min, 86% purity). MW $C_{78}H_{118}N_{22}O_{17}S$ 1666.88, measured MALDI-MS (positive) m/z = 1667.9 $[M+H]^+$, 1689.9 $[M+Na]^+$, 1705.9 $[M+K]^+$.

Synthesis of Ava-BBN2 on-resin (H_2N-Ava-Gln-Trp-Ala-Val-Sar-His-FA01010-Tle-NH-Rink Amide resin). Ava-BBN2 was synthesized according to the general peptide synthesis procedure starting from 50 mg Rink Amide MBHA resin and kept on-resin for the following dimerization with the tripeptide linker.

T*ripeptide Linker Fmoc-Ava-Lys(N_3)-Glu-OH*. Fmoc-Ava-Lys(N_3)-Glu-OH was assembled on 300 mg of the preloaded Fmoc-Glu(tBu)-Wang resin (100-200 mesh, loading: 0.57 mmol/g) according to the general peptide synthesis procedure with the *N*-terminal Fmoc group remaining attached. Release from the resin and removal of the amino acid side-chain protecting group was performed with 2 mL 95% TFA/ 2.5% H_2O/ 2.5% TIPS for 3h 20 min yielding 52 mg crude peptide. HPLC purification (Method 1: t_R = 34.5 min) and subsequent lyophilization gave the pure peptide linker as a white solid (12.8 mg, 20.6 µmol, isolated yield: 12%). MW $C_{31}H_{38}N_6O_8$ 622.28, measured ESI-MS (positive) m/z = 623.2 $[M+H]^+$.

Ava-Lys(N_3)-Glu-(Ava-BBN2)$_2$. An amount of 50 mg of Ava-BBN2 on-resin was swelled in DMF for 20 min before it was coupled with the 9.8 mg (0.4 eq., 15.7 μmol) Fmoc-Ava-Lys(N_3)-Glu-OH linker using 11.9 mg (0.8 eq., 31.4 μmol) HBTU, 4.5 mg (0.8 eq., 31.4 μmol) Oxyma, and 10.9 μL (1.6 eq., 62.8 μmol) DIPEA in 1 mL DMF for 16 h at r.t. After washing steps with DMF, the Fmoc group was removed using piperidine according to the standard peptide synthesis procedure. The peptide on-resin was treated with 2 mL of an acidic cocktail of TFA/H_2O/TIPS: 95 : 2.5 : 2.5 resulting in 14 mg of crude peptide. Semipreparative HPLC purification (Method 2: t_R = 36.1 min). HPLC solvent was reduced under vacuum using a rotary evaporator, and lyophilization gave 5.4 mg (2.2 μmol, 56% isolated yield) of the pure peptide as a TFA salt (white powder). MW $C_{120}H_{188}N_{34}O_{24}$ 2489.45, measured ESI-MS positive) m/z = 1246.1 $[M+2H]^{2+}$, 831.1 $[M+3H]^{3+}$, 623.7 $[M+4H]^{4+}$.

NOTA-Ava-Lys(N_3)-Glu-(Ava-BBN2)$_2$. An amount of 5.4 mg (1 eq.,2.2 μmol) of Ava-Lys(N_3)-Glu-(Ava-BBN2)$_2$ and 1.3 mg (1.1 eq., 2.4 μmol) of *p*-SCN-Bn-NOTA was dissolved in 100 μL of DMF in a LoBind Eppendorf tube. The pH was adjusted to 9 by the addition of 3.6 μL (26.4 μmol) of 12 eq. triethylamine (TEA). The reaction mixture was incubated at 37°C overnight before it was subjected to semipreparative HPLC purification (Method 2: t_R = 31.6 min). HPLC solvent was reduced under vacuum using a rotary evaporator, and lyophilization gave 5.4 mg (1.8 μmol, 83% isolated yield) of the chelator-modified peptide as a TFA salt (white powder). MW $C_{140}H_{214}N_{38}O_{30}S$ 2939.61, measured MALDI-MS (positive) m/z = 2942.3 $[M+H]^+$.

E5B9 (H_2N-Ser-Lys-Pro-Leu-Pro-Glu-Val-Thr-Asp-Glu-Tyr-Cys-OH) peptide. E5B9 was synthesized according to the general peptide synthesis procedure starting from 75 mg Fmoc-Cys(Trt)-preloaded Wang resin (100-200 mesh, loading: 0.60 mmol/g). Protecting group removal and release from resin was performed with 2 mL 87.5% TFA/ 5% H_2O/ 5% thioanisole/ 2.5% 2,2'-(ethylenedioxy)diethanethiol for 3h 20 min. HPLC purification (Method 3: t_R = 21.8 min) and subsequent lyophilization gave the peptide as a white solid (38 mg, 27.5 μmol, isolated yield: 61.3%). MW $C_{60}H_{93}N_{13}O_{22}S$ 1379.63, measured ESI-MS (positive) m/z = 1380.5 $[M+H]^+$, 690.9 $[M+2H]^{2+}$.

DBCO-PEG$_4$-maleimide-E5B9. An amount of 5 mg (1 eq., 3.6 μmol) of E5B9 was reacted with 2.7 mg (1.1 eq., 4.0 μmol) of DBCO-PEG$_4$-maleimide in 1 mL DMF/degassed N_2-purged PBS (pH 6.8) 1:4 for 30 min.

The reaction mixture was HPLC-purified using Method 3 (t_R = 28.9 min). Lyophilization gave 4.2 mg (2.0 μmol) of a white solid (56.8% isolated yield). HPLC-QC (Method 3: t_R = 28.9 min, purity: 97.1 %). MW $C_{96}H_{135}N_{17}O_{31}S$ 2053.92, measured MALDI-MS (positive) m/z = 2055.0 $[M+H]^+$, 2076.9 $[M+Na]^+$.

E5B9-NOTA-BBN2 monomeric TM. An amount of 3.0 mg (1 eq., 1.8 μmol) of NOTA-Ava-Lys(N_3)-BBN2 and 3.7 mg (1 eq., 1.8 μmol) of DBCO-PEG$_4$-maleimide-E5B9 were reacted in 300 μL of MeCN:water 1:1 for 1 h at r.t. in a strain-promoted azide–alkyne click (SPAAC) reaction. HPLC purification of the reaction mixture using Method 2 (t_R = 30.5 min) and subsequent lyophilization gave NOTA-E5B9-BBN2 TM as a white solid (3.3 mg, 0.9 μmol, 49.3% isolated yield). HPLC-QC (Method A-1): t_R = 18.2 min min, purity: 99.5%. MW $C_{174}H_{253}N_{39}O_{48}S$ 3720.8, measured MALDI-MS (positive) m/z 3724 $[M + H]^+$, 3788 $[M + Na + K]^+$.

E5B9-NOTA-(BBN2)$_2$ dimeric TM. An amount of 5.4 mg (1 eq., 1.8 μmol) of NOTA-Ava-Lys(N_3)-Glu-(Ava-BBN2)$_2$ and 3.8 mg (1 eq., 1.8 μmol) of DBCO-PEG$_4$-maleimide-E5B9 were reacted in 600 μL MeCN:water 1:1 for 4 h at 37°C in a strain-promoted azide–alkyne click (SPAAC) reaction. HPLC purification of the reaction mixture using Method 2 (t_R = 30.9 min) and subsequent lyophilization gave NOTA-E5B9-(BBN2)$_2$ TM as a white solid (5.6 mg, 1.1 μmol, 62% isolated yield). HPLC-QC (Method A-1): t_R = 18.6 min, purity: 99.5%. MW $C_{236}H_{349}N_{55}O_{61}S_2$ 4993.53, measured MALDI-MS (positive) m/z 4995 $[M + H]^+$, 5057 $[M + Na + K]^+$.

BBN2 dimer synthesis

Synthesis of Ava-Glu-(Ava-BBN2)$_2$ (H_2N-Ava-Gln-Trp-Ala-Val-Sar-FA01010-Tle-NH_2). Ava-BBN2 was synthesized according to the general peptide synthesis procedure starting from 50 mg of Rink Amide MBHA resin. Ava-BBN2 on resin was dimerized by coupling with 8.6 mg (0.4 eq., 23.3 μmol) Fmoc-Glu-OH, 17.7 mg (0.8 eq., 46.7 μmol) HBTU, 6.64 mg (0.8 eq., 46.7 μmol) Oxyma, and 16.3 mL (1.6 eq., 93.3 μmol) DIPEA for 16 h at room temperature. The Ava linker as Fmoc-Ava-OH was coupled to Glu-(Ava-BBN2)$_2$ after removal of the Fmoc group with piperidine using the general peptide procedure. Final acidic cleavage was performed using the general peptide procedure to give 28.7 mg of crude Ava-Glu-(Ava-BBN2)$_2$. HPLC

purification using Method 1 (t_R = 21.0 min) and isolation via lyophilization gave 13.1 mg of a white solid (5.6 μmol, isolated yield: 19.2%). MW $C_{114}H_{178}N_{30}O_{23}$ 2335.37, measured MALDI-MS (positive) m/z = 2335.8 $[M+H]^+$, 2357.8 $[M+Na]^+$.

^{68}Ga-labeling of E5B9-NOTA-BBN2 monomeric (n= 7) and dimeric (n=13) TM

Radionuclide ^{68}Ga was eluted with ~5 mL of 0.6 N HCl (trace-metal grade) from the ^{68}Ge/^{68}Ga generator via an automated GRP module (Scintomics GmbH, Fuerstenfeldbruck, Germany). The high activity fraction of the ^{68}Ga eluate (1.5 mL) was collected in the plastic reaction vessel while the rest of the radioactivity was dispensed in the 20 mL syringe of the GRP module dispenser unit. An amount of 25 μg of lyophilized E5B9-NOTA-BBN2 or E5B9-NOTA-$(BBN2)_2$ TM was reacted with 1000 μL of ^{68}Ga-$GaCl_3$ and 250 μL 4 M NaOAc buffer (pH 8.3) or 220 μL 4 M NaOAc buffer (pH 9.0) at 60°C and a pH of 4.5 for 17-20 min (for monomer) or 23-25 min (for dimer) in a LoBind Eppendorf tube. ^{68}Ga-incorporation was monitored by Radio-TLC (silica TLC plates, mobile phase: 0.1 M citric acid, R_f (free ^{68}Ga) = 0.9-1.0, R_f (E5B9-^{68}Ga-NOTA-BBN2 monomeric or dimeric TM) = 0.0-0.1). The reaction mixture was diluted with 9 mL of DI H_2O and subjected to solid-phase extraction using a Sep-Pak C18 Plus cartridge (preconditioned with 5 mL MeCN and 10 mL DI H_2O). E5B9-^{68}Ga-NOTA-BBN2 TM or E5B9-^{68}Ga-NOTA-$(BBN2)_2$ TM was slowly eluted with 1.1 mL EtOH and isolated using a rotary evaporator. For subsequent in vivo evaluation, E5B9-^{68}Ga-NOTA-BBN2 TM or E5B9-^{68}Ga-NOTA-$(BBN2)_2$ TM were reformulated in 10%EtOH/saline. Quality Control was performed using analytical Radio-HPLC at a constant flow rate of 1 mL/min and the following gradient with water/0.2% TFA as solvent A and acetonitrile as solvent B: 0−3 min 10% B, 10 min 30% B, 17 min 50% B, 23 min 70% B, and 27−30 min 90% B (t_R (^{68}Ga-NOTA-E5B9-BBN2 TM) = 18.0 min, t_R (^{68}Ga-NOTA-E5B9-$(BBN2)_2$ TM) = 18.4 min).

E5B9-^{68}Ga-NOTA-BBN2 TM: Radiochemical Purity: 95-98%. Total synthesis time: 59±10 min. Radiochemical Yield: 57±11% (d.c.).

E5B9-^{68}Ga-NOTA-$(BBN2)_2$ TM: Radiochemical Purity: 96-99%. Total synthesis time: 50±8 min. Radiochemical Yield: 46±12% (d.c.).

^{68}Ga-labeling of NOTA-Ava-Glu-(Ava-BBN2)$_2$ dimer (n=5)

An amount of 25 μg of lyophilized NOTA-Ava-Glu-(Ava-BBN2)$_2$ dimer was incubated with 1000 μL of ^{68}Ga-GaCl$_3$ and 505 μL of 2 M sodium acetate buffer (pH 8.0) at 60°C for 20-25 min (pH 4.5) in a LoBind Eppendorf tube. ^{68}Ga-incorporation was monitored by Radio-TLC (silica TLC plates, mobile phase: 0.1 M citric acid, R_f (free ^{68}Ga) = 0.9-1.0, R_f (^{68}Ga-NOTA-Ava-Glu-(Ava-BBN2)$_2$) = 0.0-0.1). The reaction mixture was diluted with 9 mL of DI H_2O and subjected to solid-phase extraction using a Sep-Pak C18 Plus cartridge (preconditioned with 5 mL MeCN and 10 mL DI H_2O). ^{68}Ga-NOTA-Ava-Glu-(Ava-BBN2)$_2$ was slowly eluted with 1.1 mL EtOH and isolated using a rotary evaporator. For subsequent in vivo evaluation, ^{68}Ga-NOTA-Ava-Glu-(Ava-BBN2)$_2$ was reformulated in 10% EtOH/saline. Quality Control was performed using analytical Radio-HPLC at a constant flow rate of 1 mL/min and the following gradient with water/0.2% TFA as solvent A and acetonitrile as solvent B: 0−3 min 10% B, 10 min 30% B, 17 min 50% B, 23 min 70% B, and 27−30 min 90% B (t_R (^{68}Ga-NOTA-Ava-Glu-(Ava-BBN2)$_2$) = 17.9 min, Radiochemical Purity: 94-98%). Total synthesis time was 43-48 min. Radiochemical Yield: 38-62% (d.c.).

Supplementary Figures

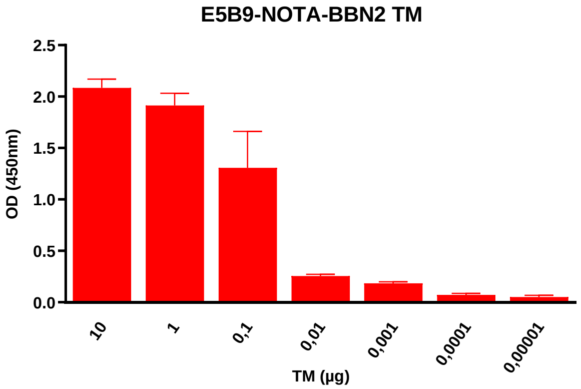

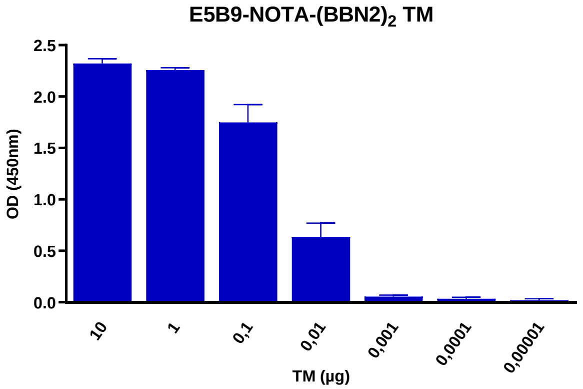

Supplementary Figure S1. Binding assessment of the 5B9 mAb to the E5B9 tag on BBN2 TMs using ELISA.

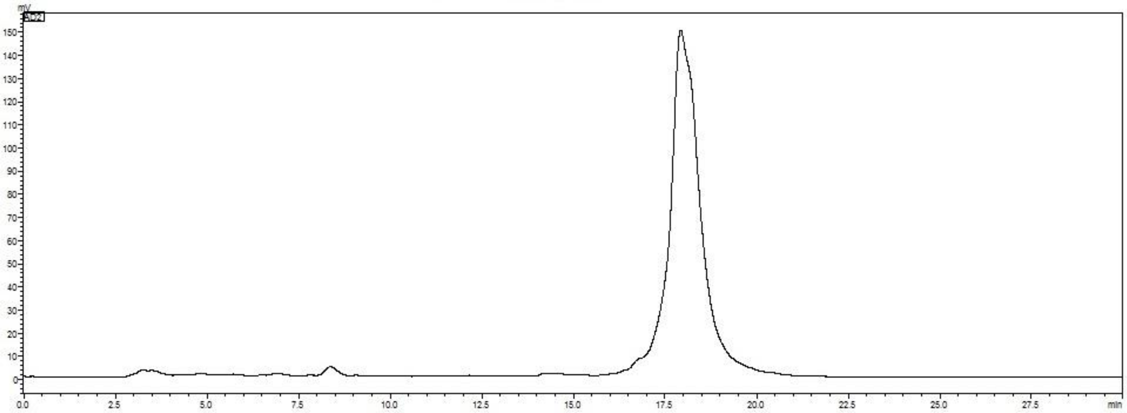

Supplementary Figure S2. Radio-HPLC-QC of E5B9-^{68}Ga-NOTA-BBN2 TM.

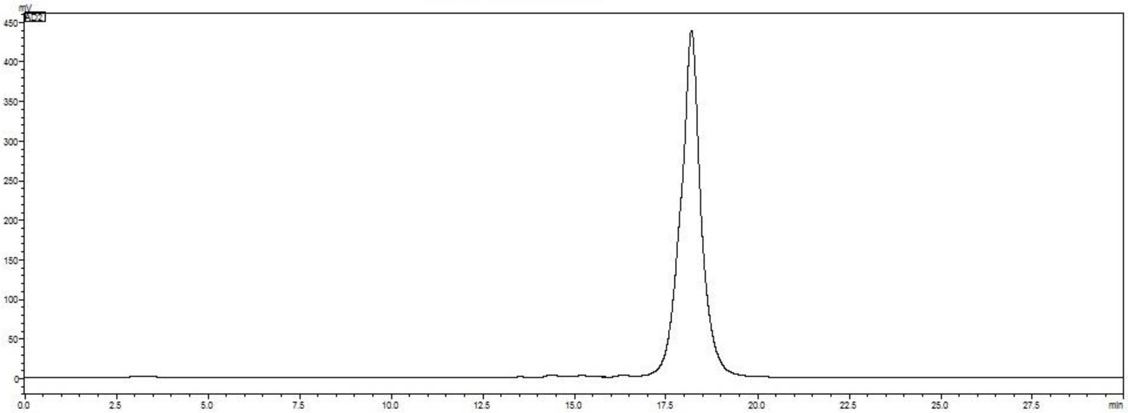

Supplementary Figure S3. Radio-HPLC-QC of E5B9-^{68}Ga-NOTA-$(BBN2)_2$ TM.

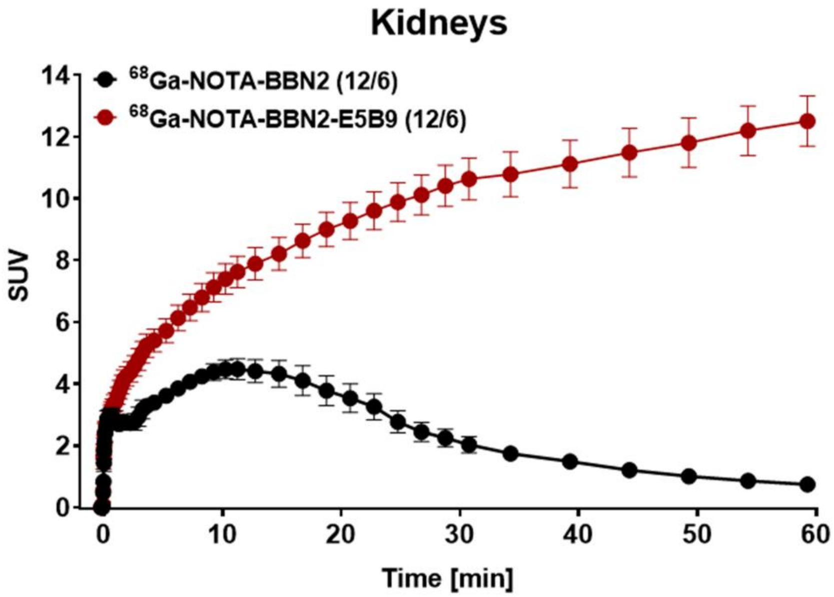

Supplementary Figure S4. Time–activity curves (TACs) from kidney clearance of ^{68}Ga-NOTA-BBN2 vs. ^{68}Ga-NOTA-BBN2-E5B9 TM in prostate cancer xenografts. Data are presented as mean ± SEM from n=6 experiments.
